# Efficacy of extra-peritoneal pelvic packing in hemodynamically unstable pelvic fractures, a Propensity Score Analysis

**DOI:** 10.1186/s13017-016-0077-2

**Published:** 2016-06-01

**Authors:** Osvaldo Chiara, Emanuele di Fratta, Anna Mariani, Bertuzzi Michaela, Lucia Prestini, Fabrizio Sammartano, Stefania Cimbanassi

**Affiliations:** SC Trauma Team, Niguarda Trauma Center, Ospedale Niguarda Ca’ Granda Milano, Milano, Italy; SC Quality Assessment Service, Ospedale Niguarda Ca’ Granda Milano, Milano, Italy; Trauma Team Ospedale Niguarda, Piazza Ospedale Maggiore 3, Milano, 20162 Italy

**Keywords:** Pelvic fracture, Hemodynamic instability, Extra-peritoneal pelvic packing, Propensity score analysis

## Abstract

**Background:**

An option for emergency control of pelvic hemorrhage is Extra-peritoneal Pelvic Packing (EPP), which addresses the retroperitoneal source of exsanguination in pelvic fractures. The aim of this study was to demonstrate the efficacy of early EPP in reducing mortality due to hemorrhage from pelvic fractures, and to evaluate the impact of packing on transfusion requirements within the first 24 h and ICU length of stay (ICU-LOS).

All data pertaining trauma patients admitted from October 2002 and December 2103 with hemodynamic instability and pelvic fractures were selected from the Hospital Trauma Registry. Patients with severe brain injury and bleeding from extra-pelvic sources were excluded. Patient population was divided into two groups: EPP group, including patients admitted from 2009 to 2013, with EPP as part of the treatment algorithm, and NO-EPP group, from 2002 to 2008, without EPP as atherapeutic option. Descriptive statistical analysis was performed on allpatients. Twenty-five patients of each group with similar features were matched using Propensity Score Analysis (PSA).

**Results:**

Six hundred eighty out of 4659 major trauma (14.6 %) presented a pelvic fracture. In 78 hemodynamically unstable patients (30 in EPP group,48 in NO-EPP group) the major source of bleeding was the pelvis. Among patients selected by PSA early mortality was significantly reduced in EPP group (20 vs 52 %, *p* = .03) compared to NO-EPP, notwithstanding similar hemodynamic impairment. No difference was observed in transfusion requirements and ICU-LOS.

**Conclusions:**

The EPP is a safe and quick procedure, able to improve hemodynamic stabilization and to reduce acute mortality due to hemorrhage in patients with pelvic fracture, in combination with optimized transfusion protocol. EPP may be useful as a bridge for time-consuming procedures, such as angio-embolization.

## Background

Hemodynamically unstable pelvic fractures still represents a diagnostic and therapeutic challenge for trauma surgeons. Pelvic ring disruption is always a marker of high energy impact mechanism, often associated with life-threatening injuries in other body districts. Despite the multidisciplinary approach mortality remains as high as 40 %, with one third of patients dying because of an uncontrolled hemorrhage [1, 2]. Bleeding in pelvic fractures originates in 85 % of cases from cancellous bone and low-pressure veins, and in the remaining 15 % from arteries [3]. Arterial bleeding may be predominant in patients with persistent hemodynamic instability after mechanical stabilization [4]. Data on pelvic injury treatments suggest that the use of angio-embolization has a success rate between 80 and 100 % in arterial bleeding control. However, this procedure is less effective when used to stop venous and bone bleeding [3]. Additionally, angio-embolization requires a lengthy set up, specialized equipment and personnel, that may not be promptly available in all hospitals. In this situation, it is not advised to transfer hemodynamically unstable patients to interventional radiology suites or to higher level facilities [5].

Extra-peritoneal Pelvic Packing (EPP) is a quick and effective procedure for the emergency control of pelvic hemorrhage that directly addresses the bleeding coming from the retroperitoneal space in severe pelvic injuries. This technique was first described in Germany in 1994 [6] and subsequently adopted by several European groups [7, 8]. In the United States, EPP was originally adopted by the Denver group and when used in association with pelvic stabilization, it showed a reduction of the mortality rate and need for transfusions and angiography [9].

The EPP is an easy and fast procedure, feasible in emergency room [7, 10]. A recent Italian Consensus Conference [8] assigned a pivotal role to EPP in the acute management of hemorrhagic shock consequent to unstable pelvic fractures, but the treatment strategy and effects on the survival still remain controversial.

The purpose of this study was to demonstrate the efficacy of early EPP in reducing the mortality due to hemorrhagic shock from pelvic fractures. The Propensity Score Analysis [11] was used to adjust the baseline characteristics and severity of different patient groups. The impact of pelvic packing on transfusion requirements within the first 24 h and ICU length of stay (ICU-LOS) were the secondary end points.

## Methods

### Patients selection

All patients in this study received a standardized protocol and were selected from the hospital Trauma Registry. Hemodynamic instability was defined as a persistent systolic blood pressure (SBP) of < 90 mmHg during initial resuscitation despite pelvic binder and ≥ 2000 ml of intravenous crystalloids and transfusion of ≥ 2 units of packed red blood cells (PRBCs). All demographic data, Injury Severity Score (ISS), Organ Injury Scale (OIS), Abbreviated Injury Scale (AIS), mechanism of injury, emergency room time, physiologic indices, lactates and base excess (BE) on admission, units of blood transfused, length of ICU-LOS, concomitant injuries, were taken into account. The pattern of pelvic fracture was classified according to Tile (A,B,C) [12] and Young & Burgess class (antero-posterior compression, APC, lateral compression, LC, vertical shear, VS, combined mechanism, CM ) [13]. Coagulation status was checked with standards exams (INR, PTT, Platelets count), while TEG/ROTEM [14, 15] was available since 2006.

Patients with pelvic fracture consecutively admitted to our level 1 Trauma Centre from October 2002 to December 2013 were selected from the hospital Trauma Registry and only those with persistent hemodynamic instability were considered for this study. The aim was to investigate the best treatment option to address bleeding exclusively or mostly caused by pelvic fracture, thus the following two categories were excluded: (a) patients with concomitant extra-pelvic bleeding requiring damage control surgery for hemorrhage and non-operated deceased patients where autopsy demonstrated a significant (about 1 L or more) extra-pelvic source of bleeding, (b) patients affected by brain injury AIS ≥ 4, deceased and non.

### Protocol of treatment

Pelvic injury patients were initially treated in our Trauma Centre by a hospital trauma team composed of a general surgeon, anesthesiologist, radiologist, radiology technician and two nurses. An orthopedic surgeon was always notified of the admission of a patient with a pelvic fracture and called to perform stabilization in emergency room and further surgical orthopedic procedures. Trauma team doctors were present in house 24 h and angio-embolization (AE) was ready available during working hours or within 60 min during weekends and nights. Hemodynamically unstable patients were transfused two 0 negative packed red blood cells followed by cross-matched packed red blood cells—fresh frozen plasma—platelets (PRBCs:FFP:PLT) at 1:1:1 ratio. Since 2008, crystalloid infusion was reduced and colloids avoided (both pre-hospital and in-hospital settings), cryoprecipitates were administered (1U/10 kg, after 20 PRBC U, or if fibrinogen < 2gr/L. This transfusion protocol did not change throughout the study period and the PRBC:FFP ratio was affected only by the overall number of PRBC (as the former were two 0 negative PRBC without FFP). Tranexamic acid (TXA) was routinely administered in class III- IV of shock only since 2011, with a loading dose of 1 gr, over 10 min, then infusion of 1 g over 8 h [16]. The source of bleeding was diagnosed with an antero-posterior chest and pelvis X-ray and Extended Focused Abdominal Sonography for Trauma (E-FAST). A total body contrast-enhanced CT-scan (CeCT) was carried out only after hemodynamic stabilization.

Before 2009 the treatment protocol of hemodynamically unstable patients with pelvic fracture consisted of: (a) temporary circumferential compression using a pelvic orthotic binder (T-POD, Bio Cybernetics International, La Verne, CA) at the level of the greater trochanters, (b) laparotomy in the operating room (OR) if E-FAST positive, (c) external fixation in the OR with the help of fluoroscopy, placed at the anterior inferior iliac spine, (d) AE if persistent instability or positive CeCT for arterial bleeding.

**Fig. 1:**
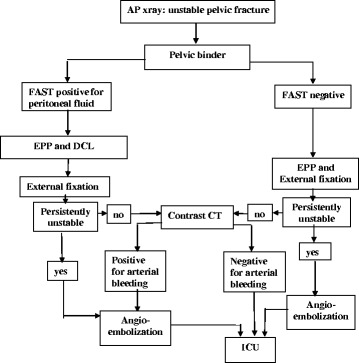
Treatment algorithm of patients with pelvic fracture and hemodynamic instability since 2009 at Niguarda Trauma Centre

After 2009 early EPP was included in the treatment options: patients with pelvic fracture and persistent hypotension despite pelvic binder and two 0-negative PRBCs would receive immediate EPP before laparotomy (if needed), followed by external fixation and AE when indicated by the presence of persistent hemodynamic instability or positive CeCT (Fig. 1).

The adopted surgical technique for EPP consisted in a vertical or supra-pubic skin incision, midline fascial cut, followed by placing 2–3 pads on each side of the bladder neck below the pelvic brim, suture of fascia and skin incision [6]. The EPP was performed directly in the emergency department (ED) inside the shock room if the patient was agonic or too unstable to be transported to the OR. The EPP was removed within 48 h in all patients.

Subjects whose hemodynamic status was stabilized by pelvic binding and 2 PRBCs transfusion underwent CeCT and AE if requested by a detectable arterial bleeding. These patients were not included in the present study.

### Statistical analysis

In this study, patients were divided in 2 groups: (a) EPP group, including patients admitted from 2009 to 2013, with EPP as a part of the treatment algorithm, (b) NO-EPP group, including patients admitted from 2002 to 2008, without EPP among the therapeutic options.

The two groups were initially compared using descriptive statistics, considering the following values: time spent in the emergency room, lactate and BE levels, age, ISS, mechanism of trauma, pelvic fracture pattern, concomitant extra-pelvic injuries, mortality with early deaths (within 24 h) and late deaths (after 24 h), transfusion of PRBCs and ICU-LOS. All patients were followed until discharge or death.

The Propensity Score Analysis was used to adjust the differences in the baseline characteristics and severity of condition at admission between the two groups. The Propensity Score was estimated modeling for potential confounders: age, ISS, pattern of pelvic fracture and non bleeding extra-pelvic injuries. With regard to exclusion criteria, only head injuries AIS 3 or less, pneumothorax, lung contusion, rupture of thoracic aorta with mediastinal hemorrhage confined, abdominal parenchymal injuries treated with non-operative management, hollow viscus injuries and fractures of the extremities were taken into consideration. A one-to-one matched analysis using nearest-neighbor matching was performed based on the estimated Propensity Score of each patient. A match occurred when one patient in the EPP group had an estimated score within 0.1 standard deviation (SD) of another in the NO-EPP group. Hence, the second study period was considered concluded when the number of EPP group was enough for the one-to-one matching with the patients selected in the NO-EPP group.

Continuous variables were compared using t-test and categorical variables with Fisher’s exact test. A *p* value of < 0.05 was considered statistically significant.

## Results

From October 2002 to December 2013, 4659 major trauma were admitted at the Niguarda Trauma Center: 680 (14.6 %) patients showed pelvic ring fractures and 111 (16.3 %) of them were hemodynamic unstable. Thirty-three patients (6 in EPP and 27 in NO-EPP group ) were excluded from the study as follows: 12 for severe traumatic brain injury AIS > 3 and 21 for extra-pelvic sources of significant bleeding. In these cases pelvic fracture was considered only partially affecting hemodynamic status and the causes of instability were identified as multifactorial. Of the remaining 78 patients, 30 were in EPP group and 48 in NO-EPP. Males were 17 (57 %) in EPP and 35 (73 %) in NO-EPP group.

All patients sustained a blunt trauma (Table 1) and common causes of pelvic fractures were falls from height, pedestrian struck and motorcycle crash. The average time inside the emergency room from the admission to the conclusion of the emergency procedures was 22 ± 8 min. in EPP and 19 ± 10 min. in NO-EPP (p ns). The age and ISS were not significantly different between groups (Table 2). Tile B1 fractures (Young & Burgess APCI/II), Tile B2 (LC I/II) and B3 (LC III) were more frequent in NO-EPP group while an increased number of Tile C fractures (Young & Burgess APCIII or VS or CM) was observed in EPP group. Extra pelvic injuries were similar between groups, in fact more than 70 % of patients were affected by injuries in other body regions. The overall mortality was higher in NO-EPP group, but not statistically significant.

**Table 1:** Mechanisms of injury in the two groups

Mechanism	EPP *n* (%)	NO-EPP *n* (%)
Car	2 (6.6)	5 (10.4)
Motorcycle	7 (23.3)	10 (20.8)
Bicycle	2 (6.6)	8 (16.6)
Pedestrian	9 (30)	10 (20.8)
Fall	10 (33.3)	13 (27.1)

**Table 2:** General characteristics of group 1 and 2 patients selected from Trauma Registry

	EPP Group (*n* = 30)	NO-EPP Group (*n* = 48)	*p*
Age (years)	55.3 ± 21.8	48.5 ± 20.8	0.1393
ISS	44.93 ± 10.06	40.57 ± 15.53	0.142
Tile Young & Burgess			0.076
A APCI-LCI	1 (2 %)	5 (10 %)	
B APCI-II, LCI-III	9 (29 %)	24 (50 %)	
C APCIII,VS,CM	20 (69 %)	19 (40 %)	
Extra-pelvic injuries	23 (77 %)	33 (70 %)	0.626
Mortality	10 (33 %)	21 (49 %)	0.092

Only 25 patients in each group resulted eligible according to the propensity Score matching. Table 3 shows the patients who were selected. Baseline characteristics were well balanced with homogeneity of potential confounders and Propensity Scores were all within 0.1 SD in the two groups. In Table 4 matched patients are compared. The BE and lactate were not different in the two groups, indicating a similar degree of hemodynamic impairment, but the admission INR was significantly lower in the EPP group. It was possible to evaluate the type of bleeding in 19 of NO-EPP and 22 of EPP patients. A prevalence of arterial bleeding was demonstrated in both groups, particularly in EPP patients. Overall mortality was significantly decreased in EPP group. Time distribution of deaths between packed and non-packed patients was different: EPP patients showed fewer early deaths and two late deaths due to organ failure while NO-EPP patients died exclusively within 24 h. In the EPP group the systolic blood pressure increased immediately after packing from 64.1 ± 15.3 to 105.5 ± 16.5 (*p* < .0001) and heart rate decreased from 119.9 ± 23.8 to 99.23 ± 20.5 (*p* < .002). In deceased patients, the elapsed time from admission to death was significantly longer in patients who received pelvic packing. Thus, the EPP group showed a decreased rate of acute deaths due to hemorrhage by more than 50 %. Moreover, patients in the NO-EPP group underwent less AE procedures than EPP patients because of the increased rate of acute mortality: eight (32 %) NO-EPP patients died before angiography and five (20 %) died despite of embolization. Conversely, in EPP patients only 3 (12 %) did not survive until angiography and 4 (16 %) died after interventional radiology. No complications related to EPP were observed.

**Table 3:** Potential confounders in patients matched with Propensity Score Analysis and calculated Propensity Scores

EPP GROUP						NO-EPP GROUP					
ID	Age	ISS	Tile/Y&B	Extra-pelvic injuries yes/no	Propensity Score	ID	Age	ISS	Tile/YB	Extra-pelvic injuries yes/no	Propensity Score
P.D.	19	50	C1/APCIII	yes	0.27	P.M.	15	68	C3/CM	Yes	0.27
C.G.	46	50	C2/VS	yes	0.52	B.E.	62	59	C3/CM	Yes	0.48
T.A.	77	41	B3/LCIII	yes	0.44	P.A.	85	50	B3/LCIII	No	0.39
A.J.	20	43	C2/VS	yes	0.44	F.S.	35	41	C2/VS	No	0.41
P.G.	60	48	C1/APCIII	yes	0.51	P.M.	38	45	C2/VS	yes	0.52
E.A.	85	38	B2/LCI	yes	0.55	B.M	46	38	C2/VS	No	0.50
P.A.	79	41	B2/LCI	yes	0.35	I.C.	52	25	B2/LCI	No	0.36
A.L.	59	45	C1/APCIII	yes	0.40	M.P.	41	25	B3/LCIII	No	0.40
V.M.	55	38	C1/APCIII	yes	0.59	F.G.	63	25	B3/LCIII	yes	0.66
S.G.	76	43	B2/LCII	yes	0.31	C.P.	42	50	B3/LCIII	yes	0.29
C.A.	63	34	C1/VS	yes	0.54	Z.F.	35	32	C2/VS	No	0.50
C.E.	84	38	B3/LCIII	yes	0.51	A.F.	36	32	C2/VS	yes	0.51
L.P.	72	50	B2/LCII	yes	0.35	F.A.	80	43	B2/LCI	No	0.34
I.A.	27	57	C1/VS	yes	0.16	L.P.	30	59	C1/APCIII	yes	0.15
P.L.	69	38	B2/LCII	yes	0.32	T.D.	38	34	B2/LCII	yes	0.33
M.P.	77	25	C2/VS	no	0.79	C.I.	65	26	C3/CM	No	0.80
B.A.	52	34	C2/VS	no	0.58	G.P.	60	59	C2/CM	yes	0.50
N.B.	69	25	B2/LCI	No	0.46	C.A.	29	59	C3/CM	yes	0.43
P.L.	70	38	C2/VS	yes	0.76	A.G	81	26	C2/VS	No	0.80
G.G.	53	25	C1/APCIII	no	0.58	G.R.	62	19	B2/LCI	No	0.48
M.L.	34	43	C2/VS	yes	0.52	M.G	79	29	B2/LCI	No	0.47
A.A.	82	38	C2/CM	yes	0.36	B.G.	83	75	C1/APCIII	yes	0.36
P.M.	80	29	C3/CM	no	0.84	C.M	88	41	C2/VS	yes	0.81
C.M.	55	25	B2/LCII	no	0.38	R.D.	40	57	C3/CM	yes	0.37
G.J.	24	41	C2/VS	yes	0.48	G.Z.	36	45	C1/APCIII	yes	0.40

**Table 4:** Comparisons of the two groups matched with Propensity Score Analysis. Continuous data are expressed as mean values ± SD

	EPP Group (25 pts)	NO-EPP Group (25 pts)	*p*
Base Excess (mEq/L)	−4.33 ± 3.78	−4.88 ± 3.38	0.29
Lactate (mmol/L)	5.18 ± 2.55	5.80 ± 3.38	0.23
INR	1.91 ± 090	1.46 ± 0.33	0.03
Arterial bleeding	22 (88 %)	19 (76 %)	-
Number of total deaths	7 (28 %)	13 (52 %)	0.01
Deaths >24 h	2 (8 %)	0	-
Deaths < 24 h	5 (20 %)	13 (52 %)	0.03
Hours from admission to death	122.8 ± 63.11	7.38 ± 10.43	0.003
n. of angio-embolizations	21 (84 %)	14 (56 %)	0.05
n. of PRBCs < 24 h	13.00 ± 11.00	14.10 ± 11.00	0.71
ICU-LOS (days)	19.80 ± 12.60	18.70 ± 11.00	0.73

There were no differences of PRBC requirement within the first 24 h after admission: packed and non-packed patients were both substantially transfused, with a mean of 14 and 13 units respectively. The PRBC:FFP ratio was 1.16 ± 0.8 in survived and 1.28 ± 0.09 (p ns) in non-survived patients. All the patients in this study were admitted to ICU and ICU LOS was not different among the two groups.

## Discussion

Despite the advances in the acute management of hemodynamic unstable pelvic fractures, the mortality rate of the affected patients is still higher than 40 % [6], with exsanguination being the main cause of acute deaths and multi-organ failure of late deaths. We hypothesized that early EPP is a “bridging treatment” in pelvic fractures with severe hemodynamic instability, useful to stabilize the source of bleeding thus increasing the safety of diagnostic and hemostatic procedures, such as ceCT-scan, angio-embolization, DCL and external fixation. Moreover, EPP may be indicated as a life-saving procedure in peripheral hospitals, before transferring the patient to higher level facilities. In our Trauma Centre patients with unstable pelvic fractures are splinted with the pelvic binder, adapted to the fracture type after initial AP x-Ray. The pelvic binder is further closed in Tile B1 (Young & Burgess APC I-II) and optimized with a posterior compression in Tile C (Young & Burgess APC III/VS/CM) fractures. The EPP is immediately performed by a trauma surgeon in the emergency room in hemodynamically unstable non-responder patients after pelvic binder and transfusion of two 0-negative PRBCs. The T-POD is left in place during EPP to avoid the displacement of pelvic fragments [17, 18]. The highly significant hemodynamic effect of EPP is demonstrated in our clinical series by the significant increase in systolic blood pressure after the maneuver.

The Italian Consensus Conference on the management of hemodynamically unstable pelvic trauma held in April 2013 gives a key role to EPP in the treatment of these patients [8]. Osborn et al. [19] in a retrospective analysis of pelvic trauma patients treated with either early angiography or direct retroperitoneal packing demonstrated that EPP significantly reduces transfusion requirements over 24 h post-intervention with no early deaths. In a review of two decades literature Papakostidis and Giannoudis [20] concluded that there is not a clear superiority of EPP over angio-embolization on patient outcome and both procedures could play a complementary role to each other. The review by Suzuki et al. [21] proposed EPP as first intervention in hemodynamically unstable patients, whereas angiography could be the first choice in stabilized patients. Tai et al. [22] in a retrospective study suggested that early EPP with subsequent angiography if needed was associated with a non-significant decrease in mortality in respect to an early angiography (from 69.2 to 36.3 %). External fixation plus EPP has been indicated by Burlew et al. [23] as optimal for life-threatening hemorrhage from unstable pelvic fracture. EPP is a level III recommendation in the guidelines from Eastern Association for the Surgery of Trauma [24], with the need of future comparative studies to define the best strategy. In a recent paper, Marzi et al. [25], suggested that angiographic embolization could be used as the first line treatment in hemodynamically stable patients with arterial blush at ceCT, while pelvic packing and mechanical stabilization are immediately carried out in hemodynamically unstable patients.

The concept of pelvic packing was originally described by Pohlmann et al. [6] in Hannover, then modified by Ertel et al. [7] in Zurich and extensively applied by Moore et al. [26] in Denver. It is a quick and easy procedure with a short learning curve and a minimal blood loss. For instance, in our series, the time required to carry out EPP was 15 min (range 8–20). It can be performed either in emergency and operating room and is especially suited for austere settings where angiography is unavailable or unable to be done expeditiously. A survey done in Wales [27], showed that only 18 % of hospitals had a formal interventional radiology service available 24 h a day, while 58 % of general surgeons and 34 % of orthopedic surgeons were able to perform EPP.

In this retrospective observational study we considered the 78 patients with hemodynamic instability and pelvic fractures admitted at the Trauma Centre of Niguarda Hospital in Milan from October 2002 to December 2013. The EPP was introduced in our hospital protocol since 2009 in addition to other techniques of hemostatic emergency procedures, such as external fixation and angio-embolization. As a randomized study was not possible for ethical and practical reasons, we used the Propensity Score Analysis to reduce potential confounders related to outcome. Propensity Score Analysis was first described by Rosembaum and Rubin in 1983 [28] and has been used increasingly in medical research [11]. The Propensity Score represents the probability (between 0 and 100 per cent) of receiving treatment A rather than B for patients in a non-randomized trial. It is based on observed baseline characteristics (potential confounder factors) and summarizes all measured confounders in a single score. Hence, Propensity Score Analysis attempts to reconstruct a situation similar to randomization.

In the present study all patients with extra-pelvic sources of bleeding and severe traumatic brain injury were excluded to avoid interference on hemodynamic status from factors other than pelvic hemorrhage. The two groups were then compared for general characteristics, such as mechanism of trauma, age, pattern of fracture, ISS, associated extra-pelvic injuries. Finally the Propensity Analysis matched 25 EPP with 25 NO-EPP patients and this “quasi-randomization” [29] pointed out the effects of early EPP in the emergency treatment of unstable pelvic fracture patients. It was evident that, given a similar hemodynamic impairment at admission, in EPP group acute deaths within the 24 h following treatment were markedly reduced, notwithstanding a higher rate of arterial bleeding.

The refinement of the transfusion protocols and expertise of the trauma team undoubtedly increased survival in the second period of the study. EPP group had a lower INR value at admission, probably due to the improved pre-hospital care (shorter time, less fluids, no colloids) in the second phase of the study. In all patients in both groups massive transfusion protocol was performed accordingly to the European guidelines [30]. The 1:1:1 ratio was always started in the emergency room after two 0 negative units since the beginning of the study. Only after 2009, in the second period of the study, TXA and cryoprecipitates were used and thromboelastometry was extensively applied for further decision making. However, EPP seemed to be very effective, as demonstrated by immediate hemodynamic improvement with increase in systolic blood pressure after packing. Most likely, the early bleeding control by EPP combined with the improved transfusion protocol contributed to the decrease of early deaths. The EPP appears to be effective in the immediate recovery of hemodynamic parameters and may be life-saving in very sick patients. The refinements of transfusion protocol can be considered of fundamental importance to recover and preserve the coagulation activity with the prevention of further bleeding in the long term.

The external fixation was always performed by orthopedic surgeons in the OR after EPP, at the end of laparotomy, if needed, and before angiography because closed bone reduction could reduce the need for embolization or, inversely, could produce new bleeding points [23]. An angiography was always performed if the patient was hemodynamically unstable after EPP and pelvic fixation. In the case of hemodynamic stabilization, even if partial, we preferred to obtain a ceCT and angiography only when ongoing arterial bleeding was detected.

Our data show that EPP did not reduce the number of transfusions needed, probably due to the different acute mortality between groups. The higher early mortality and the shorter time from admission to death in NO-EPP compared to EPP group (respectively 7.38 ± 10.43 and 122.8 ± 63.11 h) allowed less time in NO-EPP patients to receive units of blood notwithstanding a supposed higher transfusion requirement. The higher survival rate in the acute phase in EPP patients increased the risk of late multiple organ failure: two patients of this group died respectively after 43 and 53 days from injury while NO-EPP patients did not show any late mortality. This observation explains also why EPP did not reduce the mean ICU-LOS in this group.

This study presents several weaknesses. First, it is a retrospective analysis of data retrieved from a Trauma Registry. However, all patients have been prospectively included in the Registry and received two types of standardized treatments, which were compared.

Second, an inherent bias may be due to the introduction of a new technique, which can lead to a more efficient practice of this regimen.

Third, the long study period and changes in transfusion protocol represent a bias in the evaluation of EPP efficacy. However EPP is highly effective on systolic blood pressure and has an unquestionable role in this clinical series on early hemodynamic stabilization, before the effects of hemostatic resuscitation.

Finally, although the study included a small population, the patients were highly representative of the optimal treatment of pelvic hemorrhage. By using the Propensity Score Analysis we were able to match for principal confounder factors, obtaining a statistical significant result.

## Conclusions

Our data suggest that EPP is a safe and quick procedure, that can be easily performed in different hospital settings.

It can significantly decrease the mortality from hemorrhagic shock in pelvis fractures when used in combination with other emergency room and damage control resuscitation strategies.

In view of its immediate hemodynamic effect, EPP should be considered particularly in highly hypotensive patients, not responding to pelvic binder and initial resuscitation.

Increased awareness and training in techniques of emergency hemodynamic stabilization, such as pelvic binder compression, external fixation and EPP, will greatly improve the standard of treatment and care of patients affected by complex pelvic fractures.

## Abbreviations

AE, angio-embolization; AIS, Abbreviated injury scale; APC, anterior posterior compression; CM, combined mechanical; CT, computed tomography; DCL, damage control laparotomy; EPP, extra peritoneal pelvic packing; FAST, focused abdominal sonography for trauma; FFP, fresh frozen plasma; ICU-LOS, intensive care unit-lenght of stay; ISS, Injury severity score; LC, lateral compression; OIS, Organ injury scale; PLT, platelets; PRBC, packed red blod cell; PSA, Propensity Score Analysis; TXA, tranexamic acid; VS, vertical shear.
